# Integrating transcriptomics and machine learning for immunotherapy assessment in colorectal cancer

**DOI:** 10.1038/s41420-024-01934-3

**Published:** 2024-04-02

**Authors:** Jun Xiang, Shihao Liu, Zewen Chang, Jin Li, Yunxiao Liu, Hufei Wang, Hao Zhang, Chunlin Wang, Lei Yu, Qingchao Tang, Guiyu Wang

**Affiliations:** Department of Colorectal Surgery, The Second Affiliated Hospital of Harbin Medical University, Harbin, China

**Keywords:** Cancer microenvironment, Tumour immunology

## Abstract

Colorectal cancer (CRC) is a highly prevalent and lethal malignancy worldwide. Although immunotherapy has substantially improved CRC outcomes, intolerance remains a major concern among most patients. Considering the pivotal role of the tumor microenvironment (TME) in tumor progression and treatment outcomes, profiling the TME at the transcriptomic level can provide novel insights for developing CRC treatment strategies. Seventy-seven TME-associated signatures were acquired from previous studies. To elucidate variations in prognosis, clinical features, genomic alterations, and responses to immunotherapy in CRC, we employed a non-negative matrix factorization algorithm to categorize 2595 CRC samples of 27 microarrays from the Gene Expression Omnibus database. Three machine learning techniques were employed to identify a signature specific to immunotherapy. Subsequently, the mechanisms by which this signature interacts with TME subtypes and immunotherapy were investigated. Our findings revealed five distinct TME subtypes (TMESs; TMES1–TMES5) in CRC, each exhibiting a unique pattern of immunotherapy response. TMES1, TMES4, and TMES5 had relatively inferior outcomes, TMES2 was associated with the poorest prognosis, and TMES3 had a superior outcome. Subsequent investigations revealed that activated dendritic cells could enhance the immunotherapy response rate, with their augmentation effect closely associated with the activation of CD8^+^T cells. We successfully classified CRC into five TMESs, each demonstrating varying response rates to immunotherapy. Notably, the application of machine learning to identify activated dendritic cells helped elucidate the underlying mechanisms contributing to these differences. We posit that these TMESs hold promising clinical implications for prognostic evaluation and guidance of immunotherapy strategies, thereby providing valuable insights to inform clinical decision-making.

## Introduction

Colorectal cancer (CRC) ranks as the third most prevalent malignancy globally and constitutes the second leading cause of cancer-related mortality [1, 2]. The standard therapeutic approach for CRC primarily includes radical resection in conjunction with chemotherapy, immunotherapy employing immune checkpoint inhibitors (ICIs), and radiotherapy. Owing to their notably high long-term remission rates, ICIs have progressively evolved into the predominant modality for CRC treatment [3]. However, individual differences in patient responses to ICIs exist [4, 5]. Consequently, in pursuit of precision medicine for CRC, it is necessary to elucidate the mechanisms underlying patient-specific responses to ICIs.

The tumor microenvironment (TME) refers to the internal and external surroundings in which tumor cells exist; it plays a crucial role in the occurrence, progression, and metastasis of tumors. It consists of stromal and various other cells, such as cytotoxic T cells [6], dendritic cells (DCs) [7, 8], and cancer-associated fibroblasts (CAFs) [9]. The TME plays a pivotal role in determining clinical outcomes and responses to ICIs [10–14]. Traditionally, cytotoxic T cells are considered crucial anti-tumor immune cells [6]; however, their transition into exhausted T cells can potentially diminish response rates to ICIs [15]. Within the TME, tumor-infiltrating immune cells exhibit a dualistic function [12, 13]. Understanding the distinctive TME features of CRC will help formulate more precise treatment strategies.

Several gene expression-based classification frameworks have been proposed to classify CRC into subtypes with distinct molecular and clinical features [16–18], such as consensus molecular subtypes (CMS) [16] and CRC intrinsic subtypes (CRIS) [17]. Various indicators, including PD-L1 [19], CXCL9 [20], and IFN-γ [21], have been employed as predictive markers for immunotherapy effectiveness. However, these classification frameworks and indicators are yet to be validated in robust models. Accordingly, we developed an innovative classification framework that integrated CRC and TME elements to effectively stratify immunotherapy responses and discern predictive factors. Through the correlation of these classification frameworks and factors, we successfully elucidate the potential mechanisms underlying the variations in immunotherapy efficacy across CRC subtypes.

## Results

### Five tumor microenvironment subtypes of colorectal cancer were identified via unsupervised clustering

**Fig. 1:**
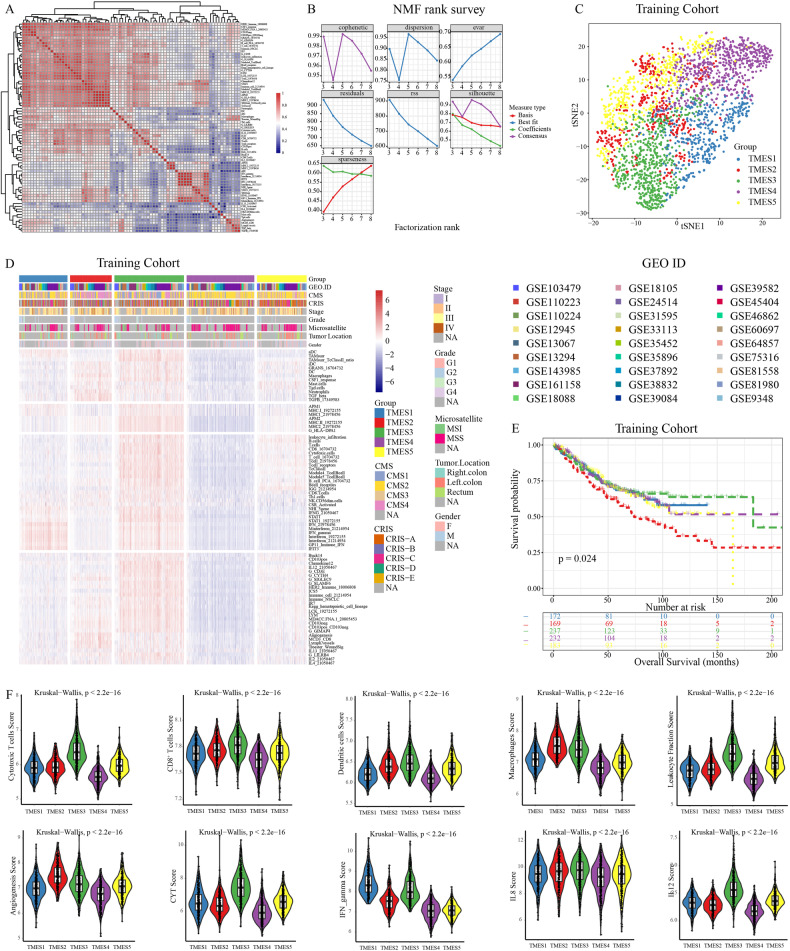
TME subtypes identified in the training cohort. **A** Correlation heatmap of 77 signatures. **B** Relationships between NMF coefficients and cluster count. **C** t-SNE projection of samples in signature space. **D** Heatmap depicting the score distribution of 2595 samples in TMESs. **E** OS Kaplan–Meier curves with different TMESs. **F** Key signature scores in TMESs.

In the training cohort, variations in specimen collection times, institutions, and sequencing platforms could introduce possible batch effects affecting real-world data accuracy. To ensure reliable results, we assessed and mitigated these batch effects in the datasets (Fig. S1A). Outlier samples within the training cohort were identified using hierarchical cluster analysis, resulting in 2595 samples after excluding 24 outliers. Signature scores were calculated by averaging the gene expression levels across 91 signatures. Using hierarchical clustering based on signature scores to filter 91 signatures, we retained 77 signatures that exhibited robust correlations (Fig. 1A). Univariate Cox proportional hazard modeling revealed an association between these signatures and CRC prognosis, with 51 signatures indicating a protective effect, whereas nine were unfavorable (Fig. S1B).

To explore differences in the TME within CRC, we utilized the NMF algorithm to identify score differences in the training cohort and stratified the patients accordingly. CRC exhibited notable differences in the TME when *k* = 5 (Fig. 1B). Consequently, the patients were categorized into five TME subtypes (TMES1–TMES5). The t-distributed stochastic neighbor embedding (t-SNE) analysis revealed a prominent distinction between TMES1 and TMES3–TMES5, whereas TMES2 exhibited extensive distribution (Fig. 1C). The heatmap further highlighted the score differences among TMESs (Fig. 1D). Similar differences in scores were observed in validation cohorts (Fig. S1C), and our submap analysis confirmed the reliability of the classification (Fig. S1D). Notably, these TMESs were associated with overall survival (OS; Fig. 1E, Fig. S1E). TMES2 was associated with the poorest prognosis, whereas TMES3 demonstrated a superior outcome, and TMES1, TMES4, and TMES5 had relatively inferior outcomes (Fig. 1E).

We also characterized the relationship between the TMESs and key signatures (Fig. 1F). TMES1 and TMES5 demonstrated an intermediate prevalence of signatures, with TMES1 showing a notable IFN-γ score. TMES2 demonstrated elevated pro-tumor signatures, including macrophages and angiogenesis scores, but lower levels of anti-tumor signatures, including cytotoxic T cell numbers, IFN-γ levels, and CYT scores, which were lower than those of TMES3. TMES3 had a significantly increased abundance of anti-tumor signatures, particularly cytotoxic T cell numbers, CD8^+^T cell numbers, IL-12 levels, and leukocyte infiltration. In contrast, TMES4 exhibited the lowest levels of various signatures, including cytotoxic T cells, leukocyte infiltration, CYT, and IFN-γ levels.

### TMESs are conserved across various cancers

**Fig. 2:**
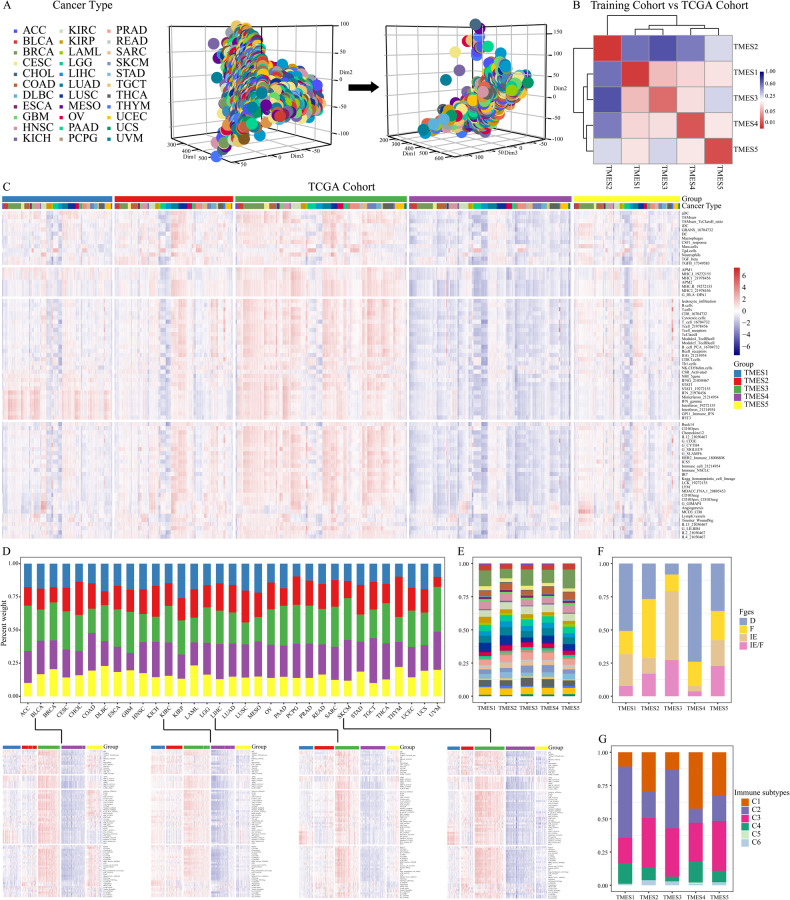
Conserved TMESs across cancers. **A** Batch effects corrected for 33 TCGA cancers. **B** Heatmap showing classification consistency between training and TCGA cohorts. **C** Signature score distribution across TMESs for >10,000 TCGA samples. **D** TMES distribution in the TCGA cohort. **E** TCGA cancer proportions in TMESs. **F**, **G** Distribution of Fges and immune subtypes in TMESs.

Transcriptomic data from pan-cancers were analyzed to validate the applicability of our classification framework across various cancers. The TCGA cohort contains over 10,000 tumor samples from 33 cancers. To account for tissue-specific effects, we eliminated them from the TCGA cohort (Fig. 2A). Our transcriptomic-based classification framework successfully helped stratify the TCGA cohort into five subtypes, closely matching the training cohort (Fig. 2B). Independent validation in a pan-cancer cohort (GSE2109), encompassing 2158 adenocarcinomas, further confirmed the robustness of the classification framework (Fig. S2A, B). Within the TCGA cohort, substantial variations in TMES scores were evident (Fig. 2C), especially for BLCA, KIRC, LIHC (liver hepatocellular carcinoma), and SKCM (Fig. 2D). Furthermore, we observed heterogeneity in the distribution of TMESs among different cancers (Fig. 2D, E).

Previous studies have established classification methods based on anti-tumor immune signatures. For example, a study employed 29 functional gene expression signatures (Fges) to create four conserved microenvironment subtypes across 24 tumors [10]. Another study categorized 33 cancers into six immune subtypes using the activity levels of five representative signatures [22]. Consequently, we investigated the correlation between TMESs and these classification methods. Notably, TMES2 and TMES4 exhibited appreciable enrichment of “F” and “D” within Fges subtypes, while TMES3 showed higher ratios of “IE/F” and “IE” (Fig. 2F). Furthermore, over 60% of TMES1 samples were categorized as “C2” in immune subtypes; TMES3 had higher proportions of “C2” and “C3,” and TMES4 was predominantly enriched “C1.” TMESs exhibited lower ratios of “C4,” “C5,” and “C6” (Fig. 2G).

Despite their presence in diverse cancers, the clinical relevance of TMESs requires further evaluation. We assessed the prognostic implications of the TMESs in both pan-cancer and individual cancers. In individual cancers, TMES2 survival was shortened (Fig. S2C), while TMES3 and TMES1 showed superior prognoses in multiple cancers (Fig. S2C). Subsequently, we conducted a comparative analysis of the prognostic impact of TMESs along with other classification methodologies. All three methodologies were associated with the OS of patients in the pan-cancer analysis (Fig. S2C–E). When analyzed for individual cancers, the TMESs were significantly associated with OS in BLCA, KIRC, LIHC, and SKCM (Fig. S2C–E).

### Characterizing clinical features of TMESs

**Fig. 3:**
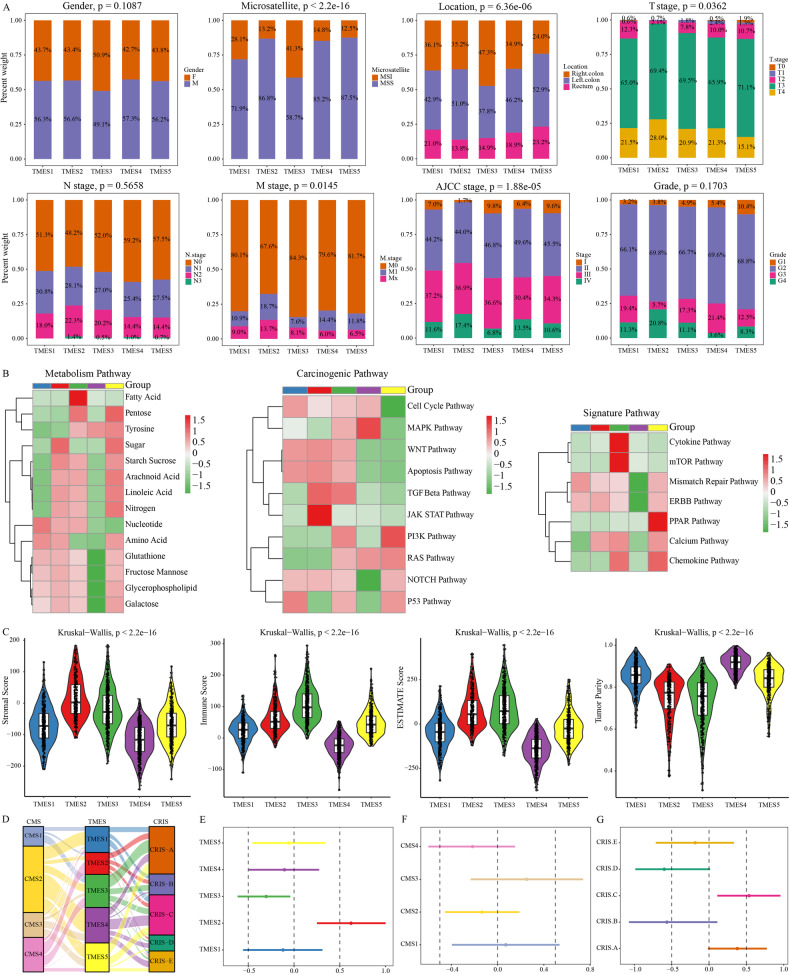
Clinicopathological variances in the training cohort. **A** Clinical distinctions across TMESs. **B** GSEA of CRC-related, metabolic, and carcinogenic pathways. **C** TME composition evaluation via ESTIMATE algorithm. **D** Sankey diagram linking TMES, CMS, and CRIS subtypes. **E**–**G** Cox proportional hazard models: log-hazard ratios and biases for TMES, CMS, and CRIS subtypes.

To infer the clinical and biological implications of TMESs, we investigated the correlations between TMESs, clinical features, and biological processes. TMES3 was more prevalent in female patients with right-sided lesions, higher histopathological grades, and higher microsatellite instability (MSI) ratios (Fig. 3A). In contrast, TMES2 was predominantly associated with left-sided lesions and advanced stages (III and IV).

We focused on transcriptomic data and performed gene set enrichment analysis, providing substantial insights into the biological understanding of TMESs (Fig. 3B). TMES1 showed strong activation of the WNT, apoptosis, P53, and mismatch repair pathways. TMES2 exhibited upregulation of metabolism-related and cancer-related pathways, especially glucose metabolism and the JAK-STAT pathway. TMES3 strongly upregulated fatty acid metabolism, cancer-related pathways, cytokine pathways, and the mTOR pathway. In contrast, most biological processes, except for the MAPK pathway, were suppressed in TMES4. TMES5 displayed remarkable activity in metabolic pathways, with upregulation of the PI3K and PPAR pathways. Additionally, we used the ESTIMATE algorithm to evaluate the TME status. TMES3 had the highest immune score and lowest tumor purity, whereas TMES2 exhibited a relatively abundant stromal composition (Fig. 3C). We also correlated TMESs with the CMS and CRIS subtypes (Fig. 3D).

Using Cox proportional hazard analyses of the training cohort, we sought to determine the potential variations in outcomes among TMESs. TMES2 was notably associated with an unfavorable prognosis, whereas TMES3 was significantly associated with a favorable prognosis. Similarly, TMES1, TMES4, and TMES5 were associated with a favorable prognosis (Fig. 3E). Importantly, TMES exhibited stronger prognostic associations than the CMS and CRIS subtypes (Fig. 3E–G), indicating their robust ability to predict CRC outcomes.

### Genomic landscape of TMESs

**Fig. 4:**
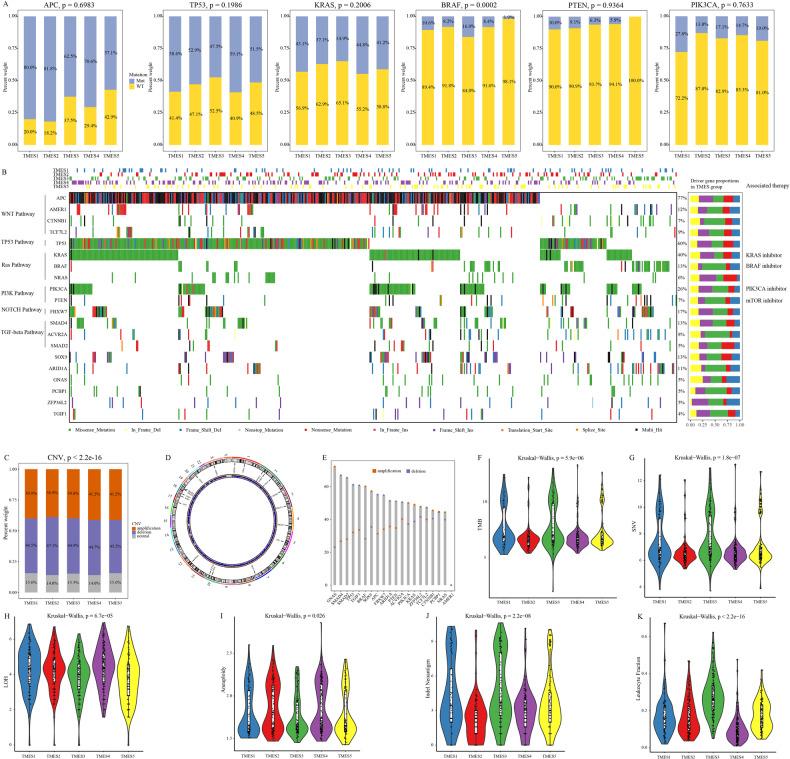
Genomic alterations in TMESs. **A**, Key driver gene mutations across TMESs in the training cohort. **B**, (left) Oncoplot of driver gene alterations in the validation cohort 1, (right) histogram of TMESs for listed alterations. **C**, CNV alterations in TMESs in the validation cohort 1. **D**, Driver gene locations on chromosomes. **E**, CNV differences in driver genes in the validation cohort 1. **F**–**K**, Comparisons of TMB, SNV, LOH, aneuploidy, neoantigen load, and leukocyte fraction.

Genomic alterations in driver genes can affect anti-tumor immunity and TME activity. Therefore, we explored the association between driver gene mutations in CRC TMESs [23]. Initially, we analyzed the association between the driver gene’s mutation frequency and TMESs (Figs. 4A and S3A). We observed higher mutation frequencies in APC, TP53, and KRAS across various subtypes. Mutations in PTEN and PIK3CA were prevalent in TMES1, whereas BRAF mutations occurred in TMES3, consistent with their known association with MSI tumors [24]. We characterized the mutation status of the driver genes in validation cohort 1 (Figs. 4B and S3B). Driver genes showed relatively high mutation ratios in TMES3, whereas only a few genes were frequently mutated (i.e., NRAS and SMAD2) in TMES2 (Fig. 4B).

Next, we investigated the relationship between CNV and TMESs in validation cohort 1. Somatic CNV did not differ between the TMESs (Fig. 4C). Figure 4D, E illustrate the chromosomal locations of driver genes and alterations in CNVs. Notably, GNAS amplification and SMAD4 deletion were the most prominent CNV alterations in driver genes (Fig. 4E). Although CNV alterations in driver genes varied across subtypes, BRAF displayed an amplified state (Fig. S3C). Moreover, we observed heterogeneity in the immunogenicity of TMESs, including TMB, SNV, LOH, aneuploidy, neoantigen load, and leukocyte fraction (Fig. 4F–K).

### Dynamic evolution of TMESs in immunotherapy

**Fig. 5:**
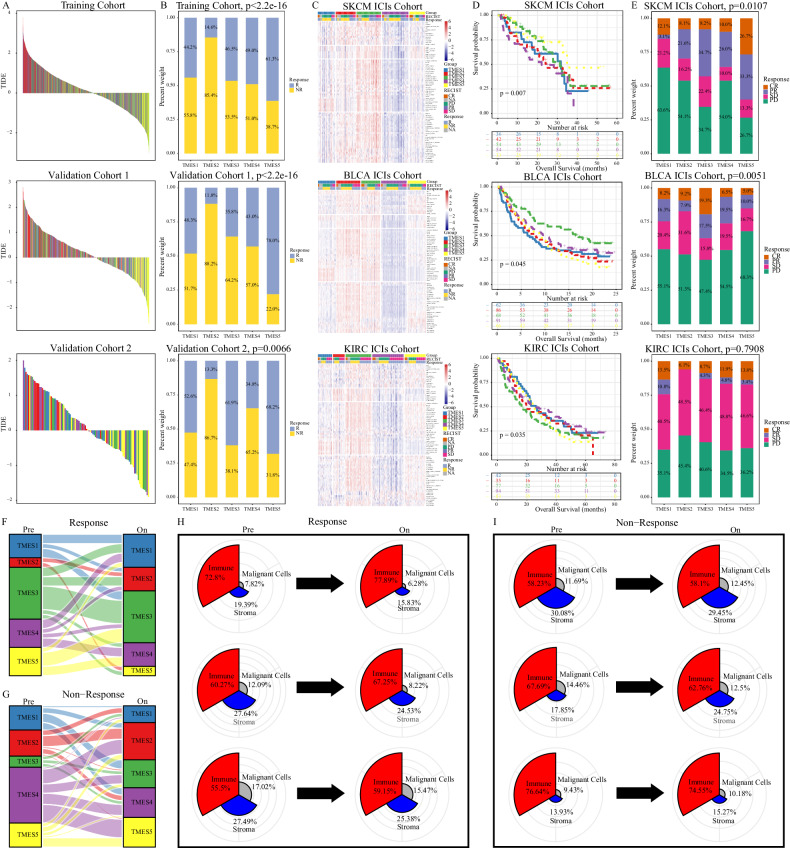
Relation of TMESs to immunotherapy. **A** Waterfall plot showing immunotherapy response correlation with TMESs in training and validation cohorts. **B** Immunotherapy response proportion in TMESs across training and validation cohorts. **C** Heatmap depicting signature score distributions in ICI cohorts (SKCM, BLCA, KIRC). **D** OS Kaplan–Meier curves for TMESs in ICI cohorts (SKCM, BLCA, KIRC). **E** Immunotherapy response ratio in TMESs in ICI cohorts (SKCM, BLCA, KIRC). **F**, **G** Pre-treatment vs. on-treatment TMESs changes in SKCM ICI cohort responders and non-responders. **H**, **I** TME composition differences pre-treatment and on-treatment in responders and non-responders in the SKCM ICI cohort.

As the TME directly affects the efficacy of ICIs, we investigated the relationship between TMESs and ICI responses to assess their potential predictive value. The TIDE scores of CRC against ICIs were assessed by utilizing the TIDE algorithm and correlating them with TMESs. Waterfall plots clearly illustrate the correlation between TIDE scores and TMESs (Fig. 5A). Patients were divided into responders and non-responders based on their TIDE scores, and the roles of TMESs in ICI responses were compared. Notably, TMES2 responders accounted for only 11.8–14.6% (Fig. 5B), while TMES5 had a higher responder proportion, in the range of 61.3–78.0% (Fig. 5B). Similar trends were observed in three independent ICI cohorts (Figs. 5C–E and S4A–C), where TMES2 had the lowest percentage of responders (6.1–29.7%, Fig. 5E), while TMES3 and TMES5 had higher percentages, ranging from 13.0–42.9 and 15.0–60.0%, respectively (Fig. 5E). These results indicate that TMES1–TMES5 can effectively predict the immunotherapy outcomes for patients.

Immunotherapy can also affect and reshape the TME. Therefore, dissecting the dynamic evolution of the TME during treatment can yield valuable insights into ICI treatment, and the SKCM ICI cohort can be analyzed to assess TME evolution. Thus, we analyzed the transcriptomic data from the SKCM ICI cohort pre- and on-treatment. In the response group, TMES3 and TMES5 emerged as the predominant subtypes, and these subtypes remained unchanged or developed into similar subtypes during treatment, whereas some non-responders to TMES2 shifted to TMES5 (Fig. 5F). In contrast, most non-responders in the non-response group remained unchanged or transitioned toward TMES2 (Fig. 5G).

We tracked the microenvironmental changes in responders and non-responders based on 29 Fges, and the results further confirmed these evolutionary patterns (Fig. 5H, I). Generally, responders exhibited an enriched immune composition and a reduced proportion of malignant cells and stroma during treatment (Fig. 5H), whereas, in non-responders, proportions of malignant cells and stroma remained constant or increased (Fig. 5I).

### Machine learning-based identification of signatures associated with immunotherapy

**Fig. 6:**
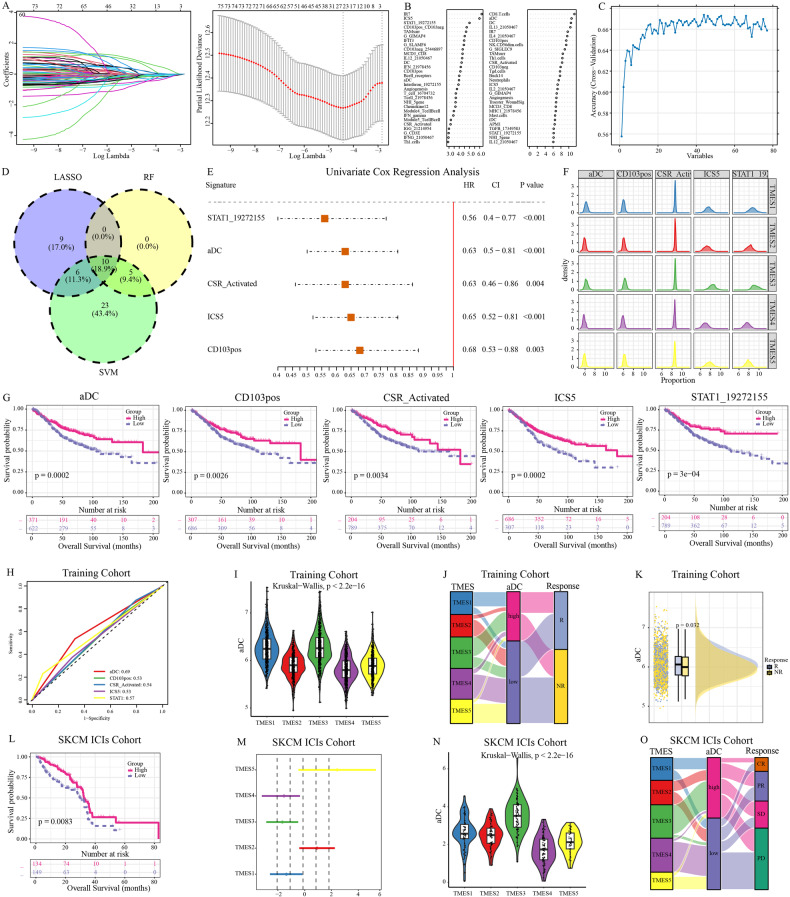
Predictive signatures for immunotherapy response. **A** LASSO-Cox regression filters signatures in the training cohort. **B** Top-30 important signatures selected via random forest. **C** SVM-RFE refines signatures. **D** Venn diagram showing signatures shared by three algorithms. **E** Forest plot of five signatures. **F** Score differences of five signatures among TMESs. **G** Survival differences with five signature cutoffs. **H** ROC curves of five signatures in the training cohort. **I** aDC score contrast in TMESs. **J** Sankey diagram linking TMESs, aDC group, and response. **K** aDC score disparity between responders and non-responders. **L** Survival contrast based on aDC cutoff in the SKCM ICI cohort. **M** log (hazard ratios) and biases for TMESs in the SKCM ICI cohort. **N** aDCs differences among TMESs in the SKCM ICI cohort. **O** Sankey diagram linking TMESs, aDC group, and response in the SKCM ICI cohort.

The TME composition is diverse, and its effects on tumors exhibit heterogeneity. To further explore the mechanisms underlying patient prognosis and differences in immunotherapy efficacy among TMESs, we integrated multiple machine learning algorithms to identify the most influential TME-related signature in CRC. We applied three machine learning algorithms, namely LASSO-Cox, RF, and SVM-RFE, to analyze the training cohort. They identified 25, 15, and 44 signatures, respectively, with 10 signatures being commonly identified across all three methods (Fig. 6A–C). We evaluated the impact of these 10 signatures on the prognosis of CRC (Fig. 6D), and the results revealed that STAT1_19272155, aDC, CSR_Activated, ICS5, and CD103pos played prominent roles in the prognosis of CRC (Fig. 6E). Through further comparison of the scores and survival differences of these five signatures within TMESs, it was found that aDCs substantially influenced the prognosis of all five TMESs (Figs. 6F, G and S5A–E). Analysis of the validation cohort supported this finding (Figure S5F–H). We assessed the predictive capabilities of five signatures using ROC curves, and the results indicated that aDCs exhibited the most outstanding predictive value (Fig. 6H).

Previous studies have indicated that DCs play an anti-tumor role by activating CD8^+^T cells through antigen presentation. aDCs represent a functional state within DCs. Therefore, we hypothesized that aDCs influence patient prognosis through immunological effects. We performed a detailed analysis of the relationship among aDCs and TMESs. The aDCs were enriched in TMES3 and TMES5 but under-expressed in TMES2 and TMES4 (Fig. 6I). Our research indicated a close association between TMESs and patient prognosis (Fig. 1E) as well as immune therapy response rates (Fig. 5B). By analyzing the immunotherapy response rates of patients with high/low-aDC groups, we found that patients in the high-aDC group were more likely to be associated with responders and TMES3, whereas those in the low-aDC group were more strongly associated with TMES2 and non-responders (Fig. 6J). Furthermore, aDCs were more abundant in responders (Fig. 6K). Similar results were observed in the melanoma ICI cohort (Fig. 6L–O). In summary, we believe that TMESs may intervene in patient prognosis by influencing the effectiveness of immune therapy through aDCs.

### aDCs cooperate with the anti-tumor function of CD8^+^T cells

**Fig. 7:**
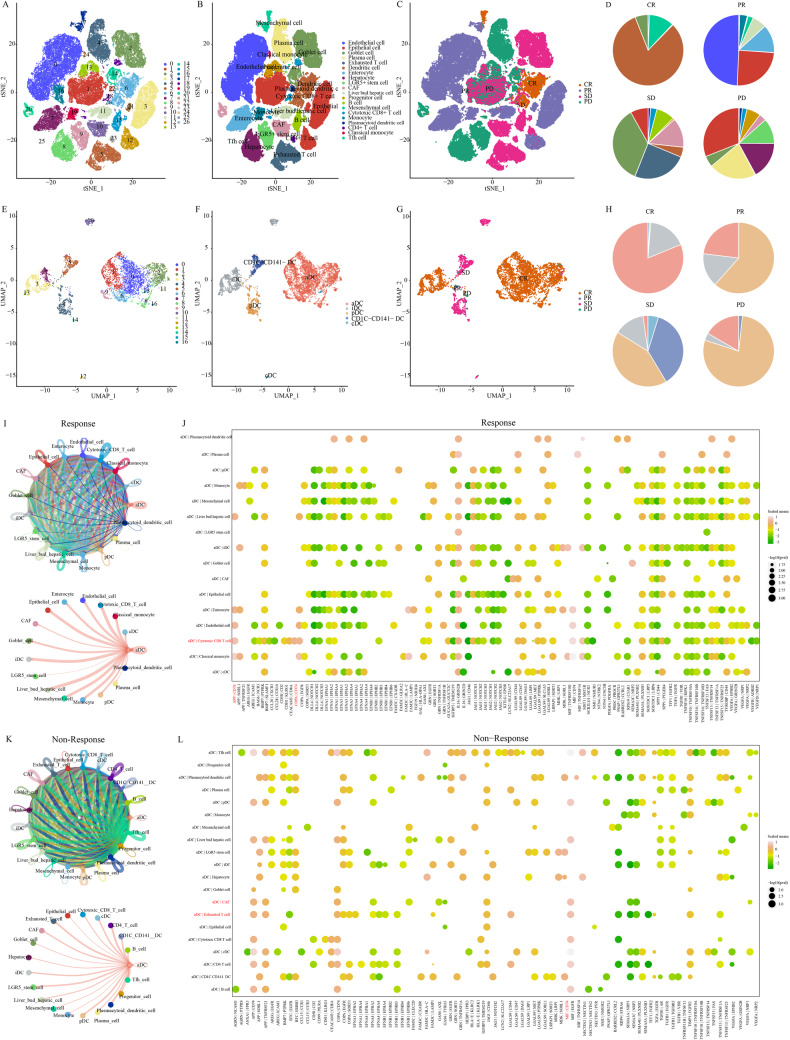
scRNA-seq data of the SCP2079 cohort illuminate the role of aDCs in immunotherapy. **A**–**C** t-SNE visualization of 88 293 high-quality cells: clusters, cell types, and response types. **D** Cell type ratios in various immunotherapy responses. **E**–**G** t-SNE of 3 162 high-quality dendritic cells: clusters, subpopulations, and response types. **H** Dendritic cell subset proportions in distinct response types. **I**–**K** aDC interaction networks in responders and non-responders. **J**, **L** Bubble plots display aDC receptor-ligand pairs with other cells in responders and non-responders.

Previous studies have suggested the potential involvement of aDCs in immunotherapy; however, the specific mechanisms remain elusive. We explored this involvement using two single-cell ICI cohorts. The SCP2079 cohort, composed of 88 293 cells, was organized into 27 clusters (Fig. 7A) representing 20 cell types (Fig. 7B). We computed the relative proportions of cell types according to the different immunotherapy responses in the ICI cohort, revealing that the responders had a high abundance of DCs (Fig. 7C, D). To evaluate the connection between aDCs and immunotherapy, we conducted a subpopulation analysis of 4829 DCs (Fig. 7E) and identified 3162 aDCs (Fig. 7F). The increase in DC numbers among responders was primarily attributed to the increased abundance of aDCs (Fig. 7G, H). These consistent trends were also confirmed in the GSE222300 cohort (Fig. S6A–F).

Further cell communication analysis revealed a prominent association between aDCs and immunotherapy. In the cell interaction network of responders, aDCs exhibited stronger associations with anti-tumor cells, such as CD8^+^T cells, whereas they demonstrated less communication with pro-tumor cells, such as CAFs (Fig. 7I). Exploring receptor-ligand interactions, we observed that aDCs and CD8^+^T cells primarily collaborated in executing anti-tumor functions through APP|CD74 and COPA|CD74 receptor-ligand pairs (Fig. 7J). In contrast, the cell interaction network of non-responders suggested that aDCs interacted more strongly with exhausted T cells and CAFs (Fig. 7K). Simultaneously, exhausted T cells and CAFs antagonized the anti-tumor functions of aDCs via the MIF | CD74 receptor-ligand (Fig. 7L). These results highlight the relevance of aDCs and immunotherapy and elucidate the underlying cell communication mechanisms that synergize or antagonize aDCs function in immunotherapy.

## Discussion

In this study, a comprehensive analysis of the genomic and transcriptomic data of CRC and the TME was conducted to classify, reconstruct, and visualize CRC composition. The study yielded four main findings: (1) it established a classification framework for CRC, dividing it into five subtypes; (2) this classification framework demonstrated pan-cancer conservativeness, making it applicable to various types of cancers; (3) the framework was effective in predicting the immunotherapy outcomes for CRC, contributing to precision medicine in CRC; and (4) the study revealed the mechanism by which aDCs promote immunotherapy through CD8^+^T cells, offering new intervention targets for subsequent research.

We performed an unsupervised analysis of transcriptomic data from 2 595 samples, identifying five TMESs that remained conserved across more than 10,000 tumor samples spanning 33 different cancers. These subtypes were closely linked to TME-associated signatures and showed a commonality in immune relationships across various cancer types. Notably, TMESs demonstrated a substantial association with OS, outperforming other classification methods such as Fges [10], immune [22], CMS [16], and CRIS subtypes [17] in their correlation with OS.

Tang et al. categorized patients with CRC into four subtypes based on immune contexts and found that these subtypes could stratify patients with different prognoses [25]. However, they did not further explore the association between these subtypes and immunotherapy. In contrast, we conducted an in-depth analysis of the relationship between TMESs and immunotherapy response rates. In our study, the differences among TMESs were also more pronounced. For example, TMES1 had the highest IFN- γ score and highly enriched PTEN and PIK3CA mutations. Notably, these tumors strongly activated the pathways associated with WNT, apoptosis, P53, and mismatch repair. These observations indicate that the TMES1 tumor subgroup is likely to respond to PI3K and mTOR inhibitors. The TMES2 tumor subgroup displayed poor prognosis, altered glucose metabolism, and activation of the JAK-STAT pathway. Particularly, TMES2 cells exhibited an extremely low response to immunotherapy. Thus, targeting the metabolic pathway (antimetabolic therapy) could be a potential therapeutic strategy for TMES2, although it requires further validation through preclinical and prospective trials [26]. TMES3 had a higher proportion of BRAF mutations and MSI and longer survival. TMES3 was particularly sensitive to immunotherapy, with upregulated fatty acid metabolism, cancer-related pathways, cytokine pathways, and mTOR pathways. These findings suggest diverse therapeutic options for TMES3. TMES4 exhibited immunosuppression and significantly reduced activity of key immune signatures such as cytotoxic T cell, and IFN-γ scores. The MAPK pathway was specifically overexpressed in TMES4, suggesting that MAPK inhibitors are likely an alternative treatment option [27]. TMES5 was associated with metabolism-related pathways and had increased activity in the PI3K and PPAR pathways. It demonstrated high sensitivity to immunotherapy, rendering TMES5 a potential therapeutic target. The TMESs were used to reclassify patients and identify novel drug-candidate targets.

Multiple studies have used machine learning models to evaluate the prognosis of patients with tumors and the efficacy of immunotherapy [28–31]. We utilized machine learning to examine immunotherapy-related signatures, elucidating the mechanisms underlying the different responses of TMESs to immunotherapy. Responders showed cooperation between aDCs and immune-promoting cells against tumors, particularly in promoting the activation of CD8+-T cells [32]. Conversely, in non-responders, the function of aDCs was inhibited by CAFs and exhausted T cells [33].

We constructed a classification system to stratify patients with CRC and compare differences in clinical outcomes and immunotherapy efficacy between patients with different cancer subtypes. The results indicated significant differences in OS and responses to immunotherapy among patients with different cancer subtypes, which may provide a reference for clinical decisions. To provide precise treatment options for patients with CRC, the patients’ gene expression profile has to be incorporated into the model to determine the cancer subtype. Based on our findings, the prognosis and proportion of response to immunotherapy are being evaluated to provide a reference for clinical decisions.

Despite providing valuable insights, our study had a few limitations. First, it relied on publicly accessible data, and further validation using real-world data is needed. Second, owing to intricate cell interactions, additional experiments are required to confirm our findings regarding aDC-CD8^+^T cell interactions. Third, previous studies have reported that neoadjuvant therapy can reduce the stage of CRC, thereby reducing local recurrence and obtaining a better prognosis. However, we only evaluated the predictive efficacy of the model for the response to immunotherapy but did not test its utility in neoadjuvant therapy. Future studies should acquire more data by conducting extensive experiments that could lead to a comprehensive understanding of TME mechanisms in immunotherapy.

In summary, we developed a classification framework utilizing TME-related signatures to categorize CRC into five subtypes. This approach has broader applications beyond CRC and reveals distinct biological processes among TMESs, offering promising therapeutic strategies for personalized treatment. Crucially, our framework can help differentiate between immunotherapy responders and non-responders. We utilized aDCs to elaborate on the underlying mechanisms that drive the diverse immunotherapy responses among TMESs. Collectively, our findings have appreciable clinical implications, aiding in prognosis assessment and clinical decision-making and highlighting aDCs as a potential therapeutic target for CRC.

## Materials and methods

### Data collection and processing

We conducted a comprehensive search across public databases (Gene Expression Omnibus [GEO, https://www.ncbi.nlm.nih.gov/geo/], cBio Cancer Genomics Portal [cBioportal, https://www.cbioportal.org/], and The Cancer Genome Atlas [TCGA, https://portal.gdc.cancer.gov/]) to acquire relevant CRC transcriptomic data. Our inclusion criteria encompassed patients that had not received chemotherapy or radiotherapy prior to surgery, specimens originating from primary CRC, datasets comprising a minimum of 1000 genes, and in CEL format. This yielded a total of 27 GEO microarrays (comprising 2619 specimens) as well as the COADREAD (colon adenocarcinoma and rectal adenocarcinoma), GSE209746, and cBioportal CRC cohorts. Additionally, pan-cancer data included GSE2109 (2158 specimens) and TCGA (11 123 specimens) cohorts. For the ICI studies, we utilized the kidney renal clear cell carcinoma (KIRC), bladder urothelial carcinoma (BLCA), and skin cutaneous melanoma (SKCM) datasets, comprising a total of 1030 patients (Tables S1 and S2).

The “affy” R package was employed to normalize CEL files within the microarrays, thereby converting probes into gene symbols. We converted ensemble IDs of RNA-seq data to gene symbols in transcripts per million (TPM) using log2(TPM+1) for normalization. The 27 microarrays constituted the training cohort, with batch effect correction executed via “sva.” The COADREAD, GSE209746, and cBioportal cohorts functioned as validation cohorts 1–3. Other datasets were testing cohorts.

Ninety-one TME-related signatures, including 65 from Wolf et al. [34], 25 from Bindea et al. [35], and exhausted T cell markers from Zhao et al. [36], were compiled by reviewing the literature (Table S3).

### Unsupervised clustering

CRC subtypes were distinguished through non-negative matrix factorization (NMF) using the “NMF” package [37]. The optimal subtype number (k) was determined based on the cophenetic correlation and dispersion coefficients of NMF; k was determined by iterating k values (3–8), applying NMF (50 iterations) for each value, and subsequently choosing the k that yielded the highest product of the coefficients. With fixed k, NMF (500 iterations) was used to define the CRC subtypes.

### Genomic data for colorectal cancer

Genomic data for CRC included somatic mutation data obtained from TCGA’s “MC3” MAF file (https://gdc.cancer.gov/about-data/publications/mc3-2017). Tumor mutation burden (TMB), single nucleotide variation (SNV), loss of heterozygosity (LOH), aneuploidy, and neoantigen load of TCGA samples were extracted from an earlier study [22]. Copy number variation (CNV) data were downloaded from the TCGA database, with amplification and deletion defined using GISTIC 2.0.

### Integrating machine learning algorithms to filter signatures

We identified signatures with superior accuracy and stability by combining support vector machine (SVM-RFE), the least absolute shrinkage and selection operator (LASSO), and random forest (RF) algorithms. SVM-RFE iteratively trims signatures with low weights in linear models while preserving those of significance. LASSO uses ten-fold cross-validation to select λ in LASSO-Cox regression, thereby eliminating low-correlation signatures. RF can help evaluate signature impact via out-of-bag (OOB) classification, Gini ranking, and accuracy, with the top-30 signatures selected based on both criteria.

### Analysis of single-cell RNA sequencing (scRNA-seq) data

The scRNA-seq data (Table S1) were processed using the “Seurat” R package [38]. Quality control involved three steps: retention of genes expressed in at least five cells, exclusion of cells expressing fewer than 100 genes, and elimination of cells expressing over 5% of mitochondrial genes. Subsequently, we normalized data using the NormalizeData function, and the top-2000 highly variable genes were identified using the FindVariableFeatures function. We used the RunPCA function to perform principal component analysis, with the top-20 principal components selected for cell clustering analysis. Differentially expressed genes in each cluster were identified using the FindAllMarkers function with threshold criteria of FDR < 0.05 and |log2 (fold change)| >0.25. Manual annotation was performed to designate cell types for each cluster.

CellPhoneDB, a publicly available resource, was employed to detect cell-to-cell receptor-ligand interactions. Using CellPhoneDB, we evaluated interaction networks by analyzing ligands, receptors, and their connections, determining interaction significance via mean expression analysis across cells.

### Statistical analysis

Statistical analyses were conducted using the R software [39]. The data met the assumptions of the tests and variation within each group of data were estimated. The variance between the compared groups is not similar. In all datasets, missing data were deleted. Two-sided tests were applied, with *P* < 0.05 being considered statistically significant. Principal component analysis was performed using the “FactoMineR” package [40], whereas linear dimensionality reduction was executed through the “Rtsne” package [41]. Survival analysis was performed using the “survminer” [42] and “survival” packages, with statistical significance evaluated using log-rank tests. The univariate Cox proportional hazard model was employed for determining hazard ratios. Additional tools employed in the analysis are listed in Table S4.

### Supplementary information

Supplementary Figures and Legends

Supplementary Table 1

Supplementary Table 2

Supplementary Table 3

Supplementary Table 4

Supplementary Table 5

## Data Availability

The primary and processed data used to generate the analyses presented here can be downloaded by registered users from GEO, cBioportal, TCGA, and supplemental material. The source data are available from the corresponding author upon reasonable request.
