# Supplementary material for: Integrating transcriptomics and machine learning for immunotherapy assessment in colorectal cancer
Source: Cell Death Discov. 2024 Apr 2;10:162. doi: 10.1038/s41420-024-01934-3 (PMC10987483; doi:10.1038/s41420-024-01934-3)
Supplement: Supplementary file 1 — Supplementary Figures and Legends [file 41420_2024_1934_MOESM1_ESM.pdf]

Supplementary Figures and Legends

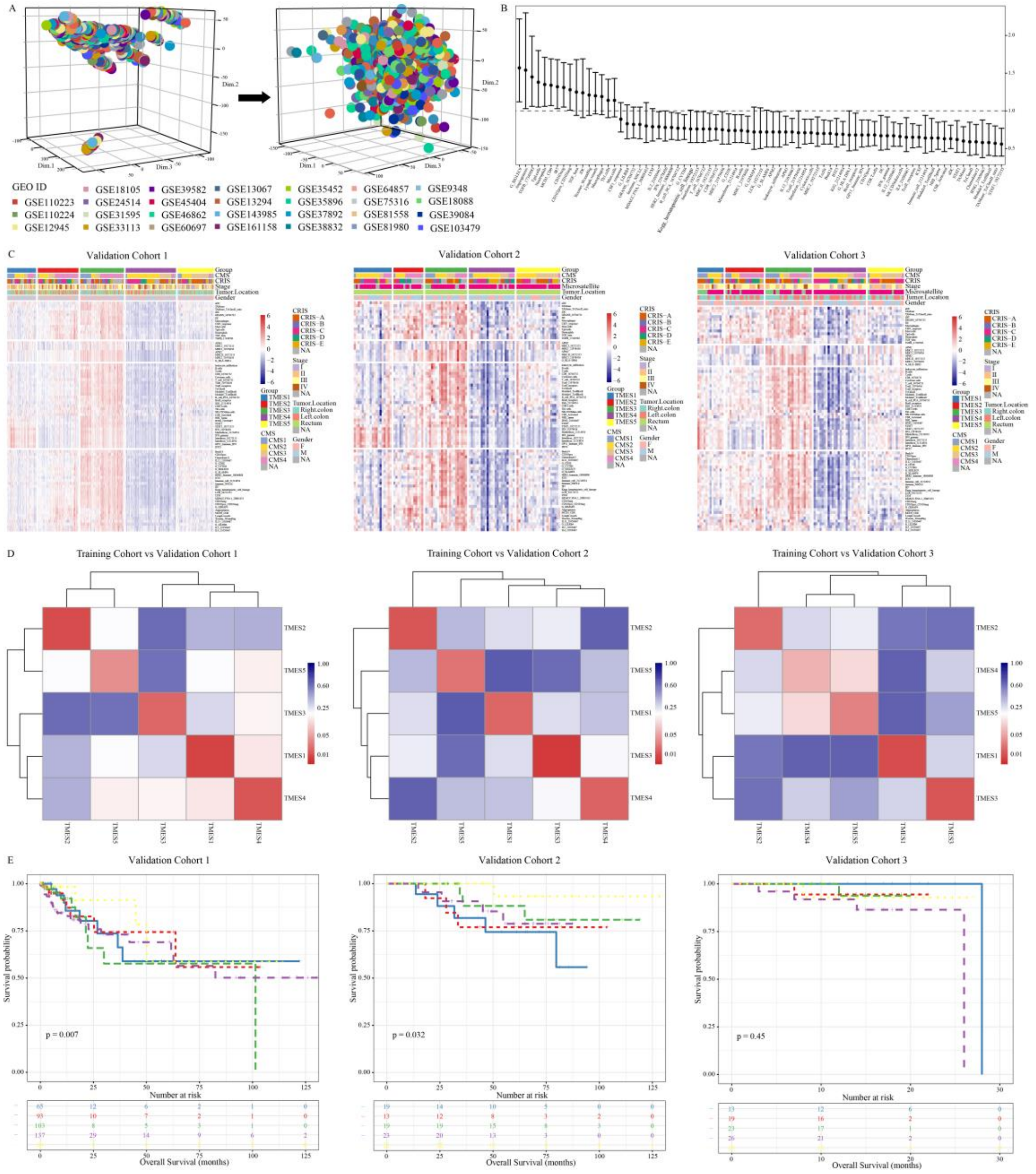

Supplementary Figure 1. TMEs in validation cohorts.

A, Batch effects correction for 27 datasets. B, Hazard ratios from univariate Cox analysis for 77 signatures. C, Heatmap of signature scores in TMES subtypes for validation cohorts 1–3. D, Heatmap of classification agreement between training and validation cohorts 1-3. E, OS Kaplan-Meier curves for different TMES subtypes in validation cohorts 1-3.

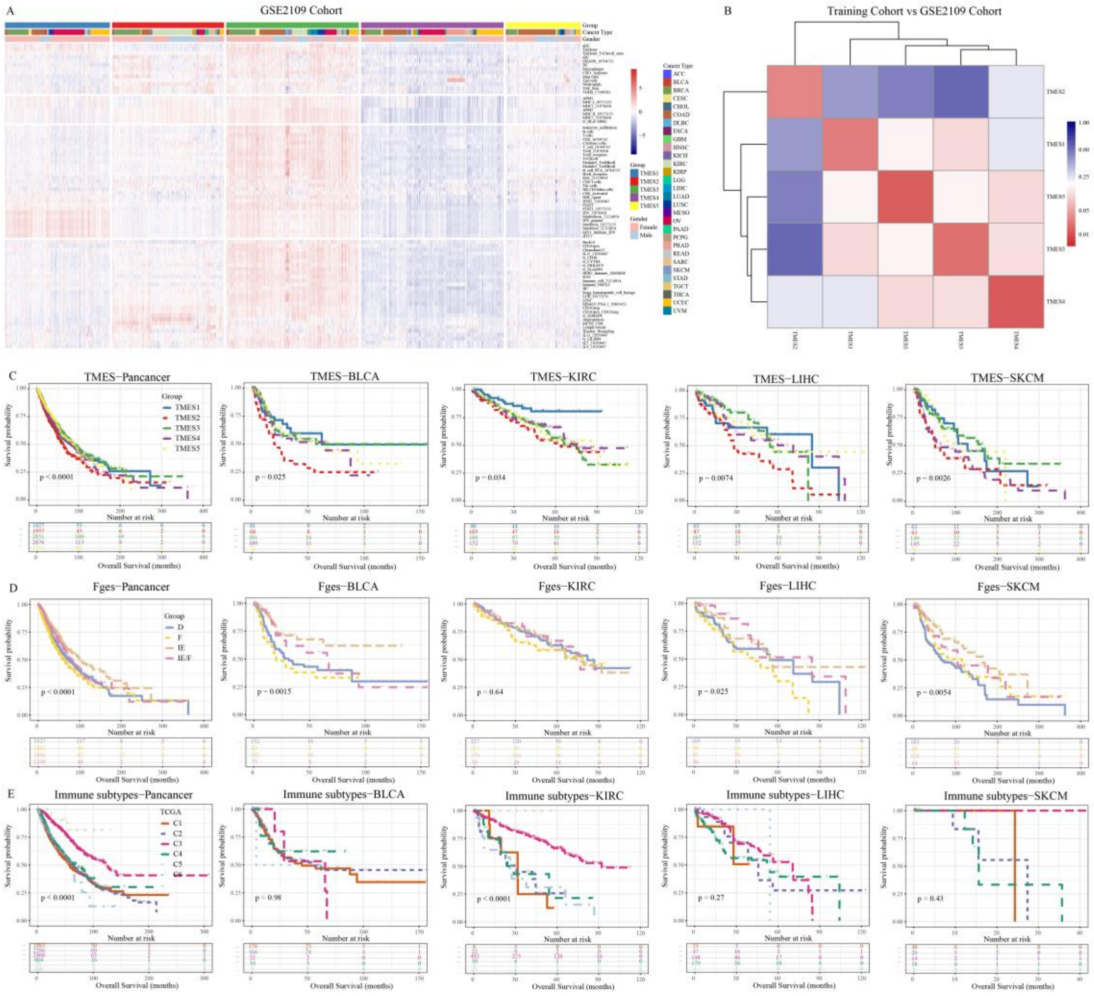

Supplementary Figure 2. Conserved TMES subtypes across cancers.

A, Heatmap of signature scores in TMES subtypes for the GSE2109 cohort. B, Classification agreement heatmap between training and the GSE2109 cohort. C, OS Kaplan-Meier curves for different TMES subtypes in the TCGA cohort. D, OS Kaplan-Meier curves for Fges subtypes in the TCGA cohort. E, OS Kaplan-Meier curves for immune subtypes in the TCGA cohort.

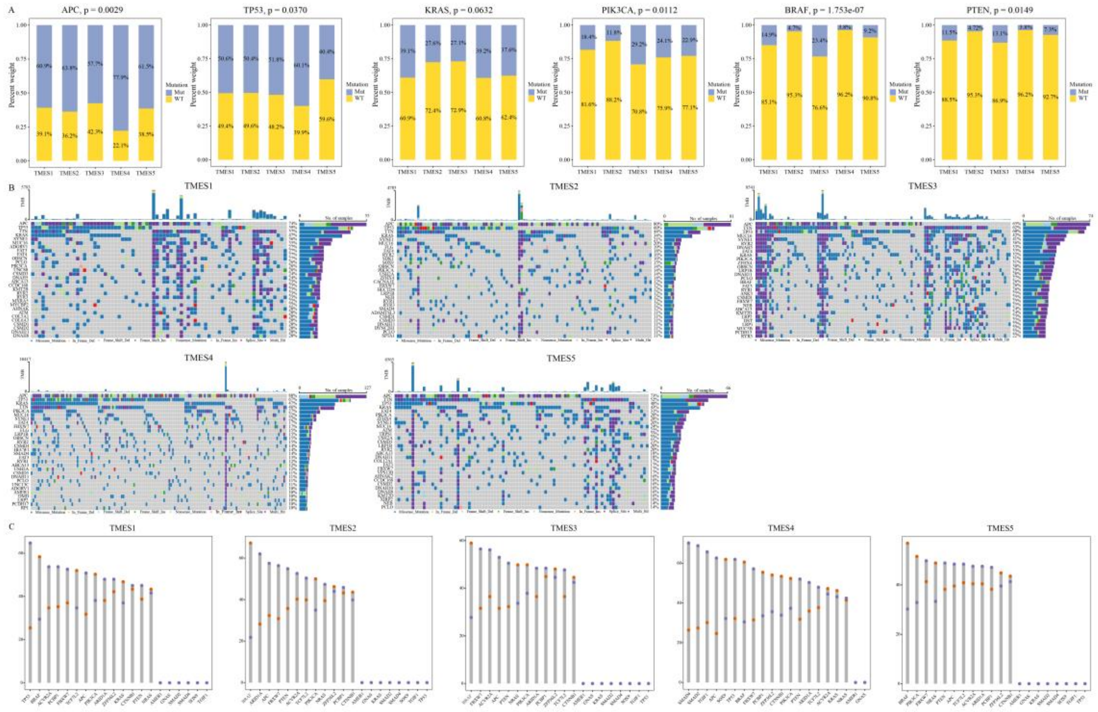

Supplementary Figure 3. Driver gene alterations in validation cohort 1.

A, Distribution of driver gene mutations in TMES subtypes. B, Oncoplot of driver gene alterations in TMES1-TMES5. C, CNV alterations comparison in driver genes of TMES1-TMES5.

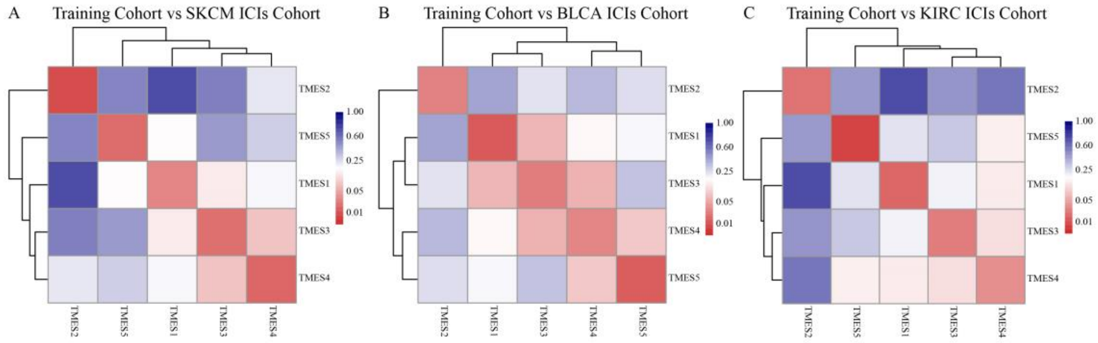

Supplementary Figure 4. Submap analysis of classification consistency in ICIs cohorts.

A, Heatmap showing training cohort and SKCM ICIs cohort classification agreement.

B, Heatmap showing training cohort and BLCA ICIs cohort classification agreement.

C, Heatmap showing training cohort and KIRC ICIs cohort classification agreement.

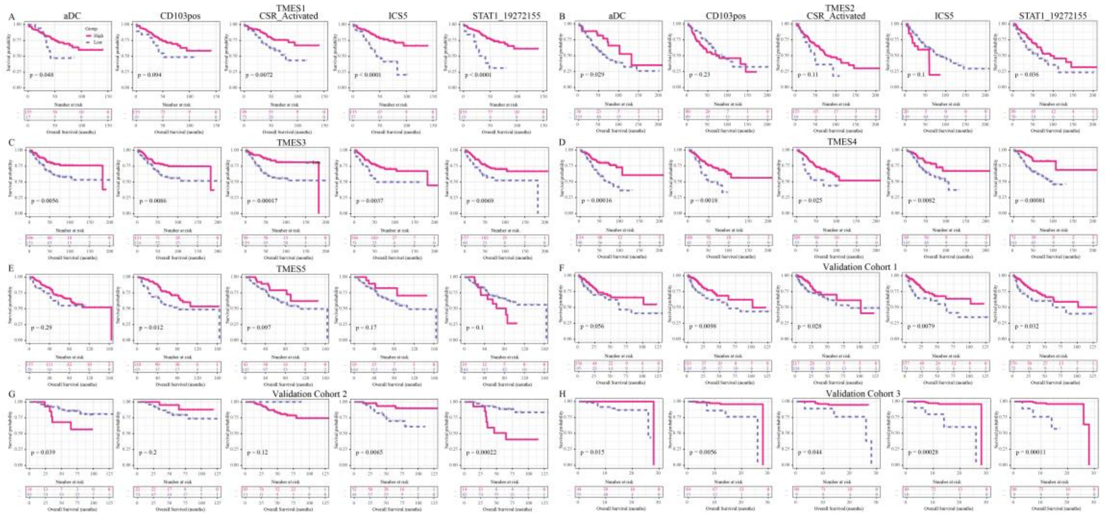

Supplementary Figure 5. Survival comparisons by signature scores.

A–E, OS differences of TMES1-TMES5 in the training cohort, using optimal aDC cutoff. F–H, OS differences in validation cohorts 1–3, using optimal aDC cutoff.

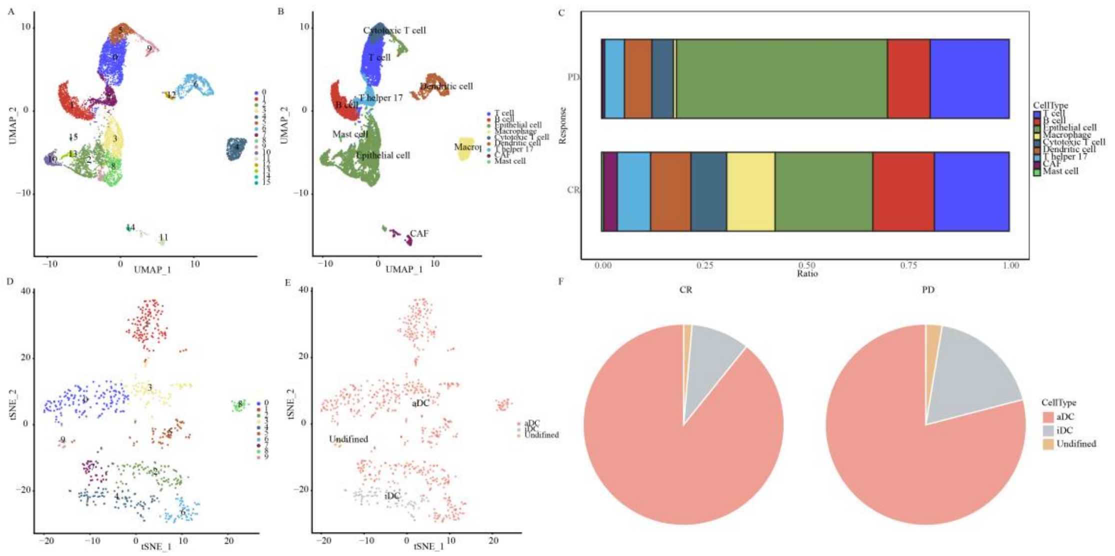

Supplementary Figure 6. scRNA-seq GSE222300 cohort validates aDCs' immunotherapy relation.

A-B, t-SNE of 8646 high-quality cells: clusters, cell types. C, Cell type ratios in diverse responses. D-E, t-SNE of 748 high-quality dendritic cells: clusters, subpopulations. F, Dendritic cell subset proportions in various responses.
