# Supplementary material for: Integrating transcriptomics and machine learning for immunotherapy assessment in colorectal cancer
Source: Cell Death Discov. 2024 Apr 2;10:162. doi: 10.1038/s41420-024-01934-3 (PMC10987483; doi:10.1038/s41420-024-01934-3)
Supplement: Supplementary file 5 — Supplementary Table 4 [file 41420_2024_1934_MOESM5_ESM.docx]

**Supplementary Table 4. The usage of tools and packages.**

| Tools/packages | Category | Version | Aims |
| --- | --- | --- | --- |
| affy[1] | R package | 1.78.0 | Processed the CEL files |
| caret[2] | R package | 6.0-94 | SVM-RFE |
| CellChat[3] | R package | 1.6.1 | Visualized cell interaction network |
| clusterProfiler[4] | R package | 4.6.2 | Enrichment analysis |
| clustree[5] | R package | 0.5.0 | Visualized clusters at different resolutions |
| CMScaller[6] | R package | 2.0.1 | Identified CMS subtypes |
| CMSclassifier[7] | R package | 1.0.0 | Identified CMS subtypes |
| CRISclassifier[8] | R package | 1.0.0 | Identified CRIS subtypes |
| estimate[9] | R package | 1.0.13 | Evaluated TME |
| ggplot2[10] | R package | 3.4.2 | Visualization of data analysis results |
| glmnet[11] | R package | 4.1-7 | LASSO-Cox |
| GSVA[12] | R package | 1.46.0 | Gene set enrichment analysis |
| limma[13] | R package | 3.54.2 | Differential expression analysis |
| maftools[14] | R package | 2.14.0 | Visualized the oncoplot |
| NMF[15] | R package | 0.26 | Identified TME subtypes |
| pheatmap[16] | R package | 1.0.12 | Drawn a heatmap |
| randomForest[17] | R package | 4.7-1.1 | Random forest |
| RCircos[18] | R package | 1.2.2 | Visualized the location of CNVs |
| Rtsne[19] | R package | 0.16 | Linear dimensionality reduction analysis of datasets |
| R[20] | Software | 4.2.3 | Bioinformatics analysis |
| Seurat[21] | R package | 4.3.0 | Single cell data processing and analysis |
| CellPhoneDB[22] | Tool | 4.0 | Identified receptor-ligand interactions between different cell types |
| GISTIC2.0[23] | Tool |  | Identified amplification and deletion of CNVs |
| Submap[24] | Tool |  | Detected consistency in subtype classification |
| TIDE[25] | Tool | 1.0 | Predicted immunotherapy response |

**Reference**

1. Gautier L, Cope L, Bolstad BM, Irizarry RA: **affy--analysis of Affymetrix GeneChip data at the probe level**. *Bioinformatics* 2004, **20**(3):307-315.

2. Kuhn MJASCL: **Caret: classification and regression training**. 2015:ascl: 1505.1003.

3. Jin S, Guerrero-Juarez CF, Zhang L, Chang I, Ramos R, Kuan CH *et al*: **Inference and analysis of cell-cell communication using CellChat**. *Nat Commun* 2021, **12**(1):1088.

4. Wu T, Hu E, Xu S, Chen M, Guo P, Dai Z *et al*: **clusterProfiler 4.0: A universal enrichment tool for interpreting omics data**. *Innovation (Camb)* 2021, **2**(3):100141.

5. Zappia L, Oshlack A: **Clustering trees: a visualization for evaluating clusterings at multiple resolutions**. *Gigascience* 2018, **7**(7).

6. Eide PW, Bruun J, Lothe RA, Sveen A: **CMScaller: an R package for consensus molecular subtyping of colorectal cancer pre-clinical models**. *Sci Rep* 2017, **7**(1):16618.

7. Guinney J, Dienstmann R, Wang X, de Reynies A, Schlicker A, Soneson C *et al*: **The consensus molecular subtypes of colorectal cancer**. *Nat Med* 2015, **21**(11):1350-1356.

8. Isella C, Brundu F, Bellomo SE, Galimi F, Zanella E, Porporato R *et al*: **Selective analysis of cancer-cell intrinsic transcriptional traits defines novel clinically relevant subtypes of colorectal cancer**. *Nat Commun* 2017, **8**:15107.

9. Yoshihara K, Shahmoradgoli M, Martinez E, Vegesna R, Kim H, Torres-Garcia W *et al*: **Inferring tumour purity and stromal and immune cell admixture from expression data**. *Nat Commun* 2013, **4**:2612.

10. Wickham HJWircs: **ggplot2**. 2011, **3**(2):180-185.

11. Tay JK, Narasimhan B, Hastie T: **Elastic Net Regularization Paths for All Generalized Linear Models**. *J Stat Softw* 2023, **106**.

12. Hanzelmann S, Castelo R, Guinney J: **GSVA: gene set variation analysis for microarray and RNA-seq data**. *BMC Bioinformatics* 2013, **14**:7.

13. Ritchie ME, Phipson B, Wu D, Hu Y, Law CW, Shi W *et al*: **limma powers differential expression analyses for RNA-sequencing and microarray studies**. *Nucleic Acids Res* 2015, **43**(7):e47.

14. Mayakonda A, Lin DC, Assenov Y, Plass C, Koeffler HP: **Maftools: efficient and comprehensive analysis of somatic variants in cancer**. *Genome Res* 2018, **28**(11):1747-1756.

15. Gaujoux R, Seoighe C: **Algorithms and framework for nonnegative matrix factorization (NMF)**. In*.*; 2010.

16. Kolde RJRpv: **Pheatmap: pretty heatmaps**. 2012, **1**(2):726.

17. Svetnik V, Liaw A, Tong C, Culberson JC, Sheridan RP, Feuston BPJJoci *et al*: **Random forest: a classification and regression tool for compound classification and QSAR modeling**. 2003, **43**(6):1947-1958.

18. Zhang H, Meltzer P, Davis SJBb: **RCircos: an R package for Circos 2D track plots**. 2013, **14**:1-5.

19. Van Der Maaten LJTjomlr: **Accelerating t-SNE using tree-based algorithms**. 2014, **15**(1):3221-3245.

20. R Core Team R: **R: A language and environment for statistical computing**. 2013.

21. Hao Y, Hao S, Andersen-Nissen E, Mauck WM, 3rd, Zheng S, Butler A *et al*: **Integrated analysis of multimodal single-cell data**. *Cell* 2021, **184**(13):3573-3587 e3529.

22. Efremova M, Vento-Tormo M, Teichmann SA, Vento-Tormo R: **CellPhoneDB: inferring cell-cell communication from combined expression of multi-subunit ligand-receptor complexes**. *Nat Protoc* 2020, **15**(4):1484-1506.

23. Mermel CH, Schumacher SE, Hill B, Meyerson ML, Beroukhim R, Getz G: **GISTIC2.0 facilitates sensitive and confident localization of the targets of focal somatic copy-number alteration in human cancers**. *Genome Biol* 2011, **12**(4):R41.

24. Hoshida Y, Brunet JP, Tamayo P, Golub TR, Mesirov JP: **Subclass mapping: identifying common subtypes in independent disease data sets**. *PLoS One* 2007, **2**(11):e1195.

25. Jiang P, Gu S, Pan D, Fu J, Sahu A, Hu X *et al*: **Signatures of T cell dysfunction and exclusion predict cancer immunotherapy response**. *Nat Med* 2018, **24**(10):1550-1558.
